# SGO1 is involved in the DNA damage response in MYCN-amplified neuroblastoma cells

**DOI:** 10.1038/srep31615

**Published:** 2016-08-19

**Authors:** Yuko Murakami-Tonami, Haruna Ikeda, Ryota Yamagishi, Mao Inayoshi, Shiho Inagaki, Satoshi Kishida, Yosuke Komata, J K Jan Koster, Ichiro Takeuchi, Yutaka Kondo, Tohru Maeda, Yoshitaka Sekido, Hiroshi Murakami, Kenji Kadomatsu

**Affiliations:** 1 Department of Molecular Biology, Nagoya University Graduate School of Medicine, 65 Tsurumai-cho, Showa-ku, Nagoya, 466-8550, Japan; 2 Division of Molecular Oncology, Aichi Cancer Center Research Institute, 1-1 Kanokoden, Chikusa-ku, Nagoya, 464-8681, Japan; 3 College of Pharmacy, Kinjo Gakuin University, 2-1723, Omori-cho, Moriyama-ku, Nagoya, 463-8521, Japan; 4 Department of Oncogenomics, Academic Medical Center, University of Amsterdam, Meibergdreef 9 1105 AZ Amsterdam, the Netherlands; 5 Department of Computer Science, Nagoya Institute of Technology, Gokiso-cho, Showa-ku, Nagoya, 466-8555, Japan; 6 Department of Epigenomics, Nagoya City University Graduate School of Medical Sciences, 1 Kawasumi, Mizuho-cho, Mizuho-ku, Nagoya, 467-8601, Japan; 7 Department of Cancer Genetics, Program in Function Construction Medicine, Nagoya University Graduate School of Medicine, 65 Tsurumai-cho, Showa-ku, Nagoya, 466-8550, Japan; 8 Department of Biological Science, Faculty of Science and Engineering, Chuo University, 1-13-27 Kasuga, Bunkyo-ku, Tokyo 112-8551, Japan

## Abstract

Shugoshin 1 (SGO1) is required for accurate chromosome segregation during mitosis and meiosis; however, its other functions, especially at interphase, are not clearly understood. Here, we found that downregulation of SGO1 caused a synergistic phenotype in cells overexpressing MYCN. Downregulation of SGO1 impaired proliferation and induced DNA damage followed by a senescence-like phenotype only in MYCN-overexpressing neuroblastoma cells. In these cells, SGO1 knockdown induced DNA damage, even during interphase, and this effect was independent of cohesin. Furthermore, MYCN-promoted *SGO1* transcription and *SGO1* expression tended to be higher in MYCN- or MYC-overexpressing cancers. Together, these findings indicate that SGO1 plays a role in the DNA damage response in interphase. Therefore, we propose that SGO1 represents a potential molecular target for treatment of *MYCN*-amplified neuroblastoma.

Shugoshin 1 (SGO1) is required to ensure the accuracy of chromosome segregation during mitosis and meiosis^1^. During Meiosis I in fission or budding yeast, Sgo1 protects pericentromeric cohesin for cleavage from separase, a role assigned to Sgo2 in mammalian cells. During mitosis in vertebrate cells, Sgo1 protects pericentromeric cohesin from removal from the prophase pathway, thereby protecting the cell from untimely sister chromatid separation^2^. The human genome encodes two Shugoshin paralogs, SGO1 and SGO2, which function during chromosome biorientation by promoting recruitment of proteins involved in the correction of erroneous kinetochore–microtubule attachments, such as the chromosomal passenger complex (CPC) and mitotic centromere-associated kinesin (MCAK)^3^. SGO1 expression is regulated by YAP/TAZ/TEAD (i.e., the Hippo pathway) and AP-1 (activator protein-1, a dimer of JUN and FOS proteins)^4^. To date, no other regulators of *SGO1* transcription have been reported. SGO1 also plays important roles in various cancers^5^,^6^,^7^,^8^,^9^; in particular, defects in SGO1 induce premature chromosome segregation, followed by chromosomal instability (CIN). The molecular mechanism underlying CIN involves dysfunction of the inner centromere–Shugoshin (ICS) network, which coordinates sister chromatid cohesion and kinetochore–microtubule attachment^10^. However, the role of SGO1 during interphase in cancer cells in general, and in neuroblastoma in particular, remains unclear.

The cohesin complex, which contains Structural maintenance of chromosome 1A (SMC1A), SMC3, RAD21, and Stromal antigen 2 (STAG2), forms a ring-like structure that holds sister chromatids together^11^. Cohesin is involved in DNA replication via interaction with minichromosome maintenance (MCM) proteins that stabilize chromatin loops and regulate the frequency of origin firing^12^. In human cells, cohesin is also involved in DNA repair: it is recruited by RAD50–MRE11 to DNA double strand break (DSB) sites after irradiation and facilitates homologous recombination (HR) by holding sister chromatids together^13^. Cohesin also plays other important roles. For example, in ES cells, cohesin, Mediator, and Nipbl regulate transcription by forming DNA loops that bring enhancers and promoters closer together^14^. Furthermore, cohesin mutations have been detected in various cancers, including colorectal cancer, glioblastoma, Ewing’s sarcoma, melanoma, and acute myeloid leukemia (AML). These mutations promote tumorigenesis by inducing genome instability due to defects in DNA replication and DNA damage repair, as well as chromosome mis-segregation^11^.

MYCN is a MYC family protein and neural tissue-specific transcription factor that contains a β-helix-loop-helix domain^15^. The MYC-binding DNA sequence motif, known as the E-box (CANNTG)^16^, is present in the promoters of many target genes, including some that encode DNA damage response (DDR) proteins^17^,^18^,^19^,^20^,^21^.

Although MYCN cannot transform cells on its own^22^,^23^, it is associated with the malignant phenotype of several human malignancies. *MYCN* is amplified in ~25% of cases of neuroblastoma, the most common extracranial solid tumor seen during childhood, and *MYCN* amplification correlates with poor prognosis. Because MYC or MYCN is required for fundamental cellular processes, MYC or MYCN inhibitors may cause undesirable side effects. Identifying the gene(s) which shows synthetic (dosage) lethal interactions^24^ with MYCN or MYC amplification may help the development of promising strategies for the treatment of MYCN- or MYC-driven cancers because inhibiting genes that show synthetic lethality with MYC or MYCN amplification would selectively kill cancer cells^25^,^26^,^27^,^28^,^29^,^30^,^31^,^32^,^33^,^34^,^35^,^36^.

We previously reported that the condensin subunit SMC2 is a target of MYCN, and that SMC2 downregulation causes a synergistic phenotype in conjunction with MYCN amplification or overexpression^35^. In that study, we showed that SMC2 regulates transcription of DDR genes in cooperation with MYCN.

Here, we demonstrate that MYCN overexpression/amplification and SGO1 knockdown synergistically inhibit cell proliferation. The growth defect in SGO1-knockdown/MYCN-overexpressing/amplified cells is the result of persistent DNA damage, which leads to a senescence-like phenotype. In MYCN-overexpressing neuroblastoma cells, SGO1 knockdown induced DNA damage even in interphase, and this phenotype was independent of cohesin. In addition, we found that *SGO1* is a transcriptional target of MYCN, and that SGO1 expression correlates with MYCN or MYC expression in various cancers. These results suggest that SGO1 represents a potential molecular target for therapeutics against MYCN- or MYC-overexpressing cancers.

## Results

### SGO1 expression is elevated in MYCN- or MYC-overexpressing cancers and cell lines

**Figure 1:**
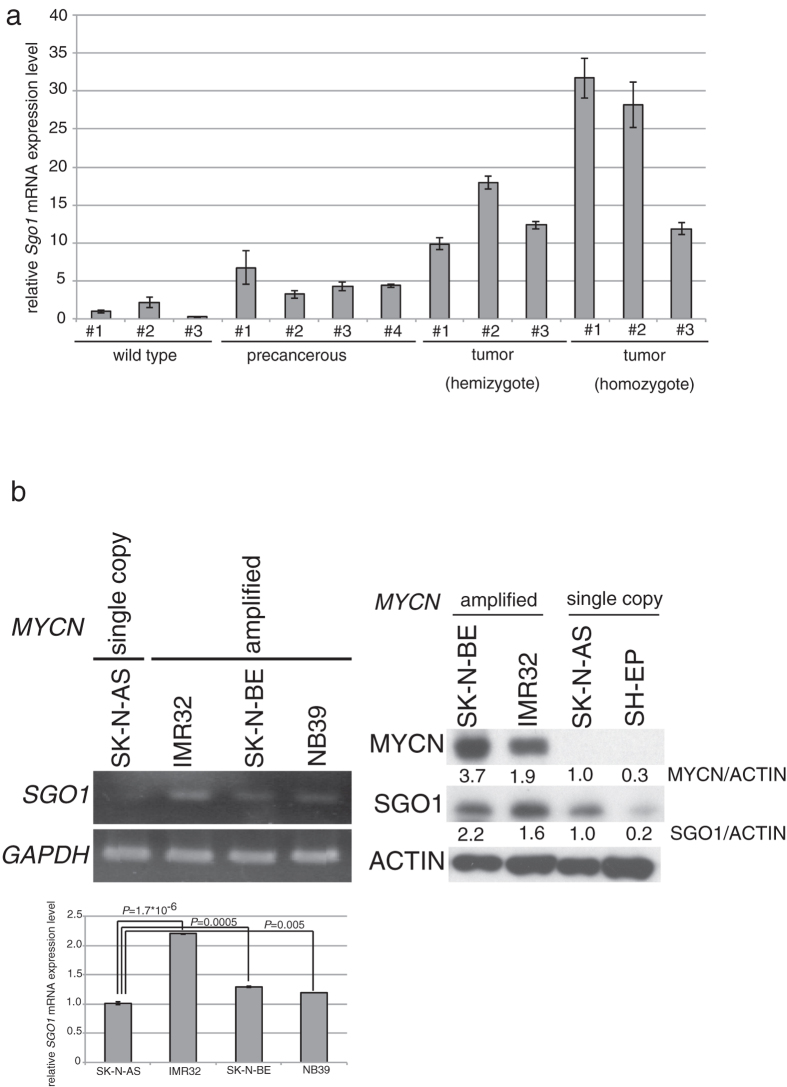
*Sgo1* expression increases with neuroblastoma progression, and *SGO1* expression is elevated in *MYCN*-amplified neuroblastoma cell lines. (**a**) Quantitative RT-PCR analyses of *Sgo1* mRNA levels in precancerous lesions from four hemizygous *MYCN*-Tg mice (1.6 or 2 wks old), tumor lesions from three hemizygous MYCN-Tg mice (9 or 10 wks old) and three homozygous *MYCN*-Tg mice (6 or 6.1 wks old), and ganglia from three wild-type mice (1.6 or 2 wks old). (**b**) SGO1 mRNA (left upper : semi-quantitative, left bottom : quantitative qPCR analysis) and protein (right) levels in neuroblastoma cell lines.

In a previous study, we used microarray data (GEO accession: GSE43419) to identify genes induced during progression of neuroblastoma in *MYCN*-Tg mice^35^. Among these genes, we focused on *Sgo1* (Fig. S1a). To confirm the microarray results, we performed quantitative RT-PCR on RNA from ganglia of wild-type (wt), hemizygous, and homozygous *MYCN*-Tg mice (Fig. 1a). As expected, *Sgo1* mRNA levels in precancerous and tumor samples were high. Next, we measured *SGO1* expression in neuroblastoma samples from patients (GSE19274) using the R2 bioinformatics platform (http://r2.amc.nl). Consistent with the expression pattern in *MYCN*-Tg mice, *SGO1* expression was elevated in human *MYCN*-amplified tumors (Fig. S1b). Likewise, SGO1 mRNA and protein levels were higher in *MYCN*-amplified neuroblastoma cell lines (IMR32, SK-N-BE, and NB39) than in a neuroblastoma cell line harboring a single copy of MYCN (SK-N-AS) (Fig. 1b). Based on these findings, we investigated the relationship between MYCN or MYC and SGO1/SGO2 expression levels using The Cancer Genome Atlas (TCGA) pan-cancer gene expression datasets (Fig. S1c). Expression of MYCN correlated with that of SGO1/SGO2, except in lower-grade gliomas, and SGO1/SGO2 expression tended to be higher in some MYC-overexpressing cancers. Together, these results showed that SGO1 is elevated in MYCN- or MYC-overexpressing cancers, including neuroblastoma cell lines showing MYCN amplification.

### *SGO1* is a potential novel transcriptional target of MYCN

**Figure 2:**
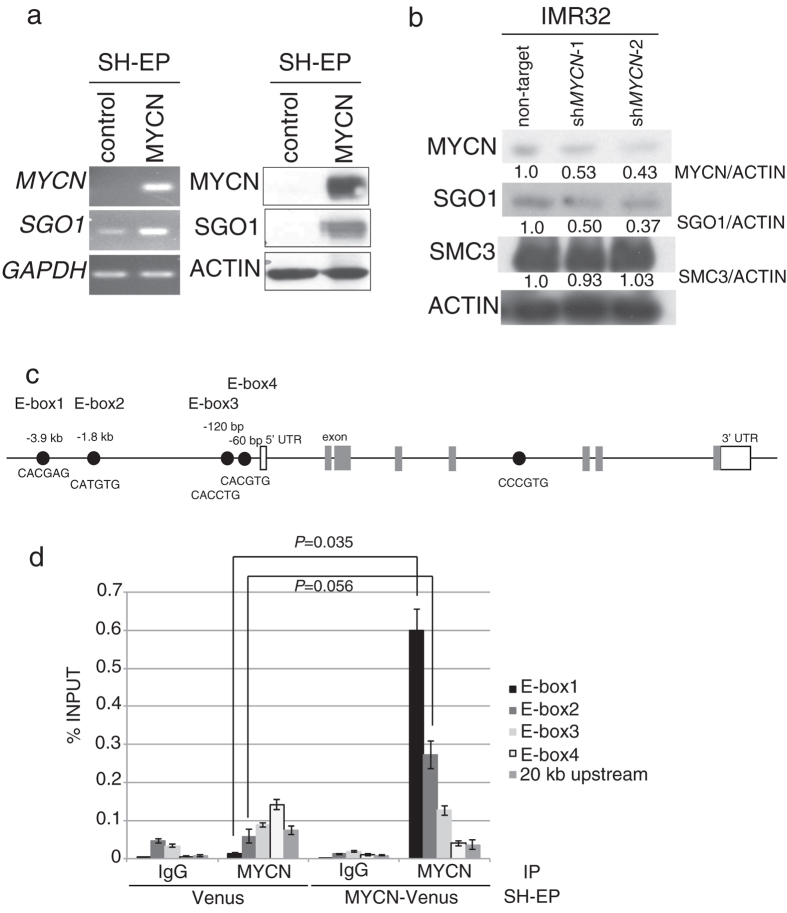
*SGO1* is a potential transcriptional target of MYCN. (**a**) MYCN overexpression induced *SGO1* mRNA (left) and protein (right) expression in SH-EP cells (*MYCN* single-copy neuroblastoma cell line). (**b**) SGO1 protein levels were reduced in MYCN-downregulated IMR32 cells, but the SMC3 protein level was unchanged in these cells. Samples were harvested 3 days after non-target or MYCN-targeted shRNA lentivirus infection. Quantification of proteins on Western blots using ImageJ software. (**c**) Positions of E-box sequences associated with *SGO1*. Black circles, E-boxes; white boxes, 5′- or 3′-UTRs; gray boxes, exons. (**d**) ChIP analysis revealed enrichment of MYCN in E-box1 and E-box2 upstream of *SGO1*. A sequence 20 kb upstream of the *SGO1* gene was used as a negative control. Data show the percentage of target DNA precipitated with control IgG or MYCN antibody, and are expressed as the means ± SE of at least three independent experiments.

To determine whether MYCN regulates *SGO1* mRNA levels, we measured changes in SGO1 mRNA levels using SH-EP cells harboring a single copy of MYCN. MYCN overexpression induced SGO1 upregulation at both the mRNA and protein levels (Fig. 2a). In addition, SGO1 protein levels fell when MYCN was downregulated in IMR32 cells (Fig. 2b). Since MYC family transcriptional factors bind E-boxes, we searched for the latter within the SGO1 genome sequence and found four (E-box1–4) in the 4 kb region upstream of the *SGO1* start codon and one in a *SGO1* intron (Fig. 2c). To determine whether MYCN binds to the E-box sequences upstream of *SGO1*, we next performed chromatin immunoprecipitation (ChIP) assays. ChIP analysis revealed that MYCN associated most strongly with E-box1 (*P* = 0.035), and tended to associate with E-box2 (*P* = 0.056). It also associated with E-box3, but much less strongly (Fig. 2d). The association between MYCN and E-box4 was minimal, and was essentially no stronger than its association with the genomic region located >20 kb upstream of the *SGO1* start codon used as a negative control. These results indicated that *SGO1* is a potential novel transcriptional target of MYCN through binding to E-box1 and E-box2.

### SGO1 downregulation impairs cell proliferation and causes MYCN-overexpressing neuroblastoma cells to accumulate at G2/M

**Figure 3:**
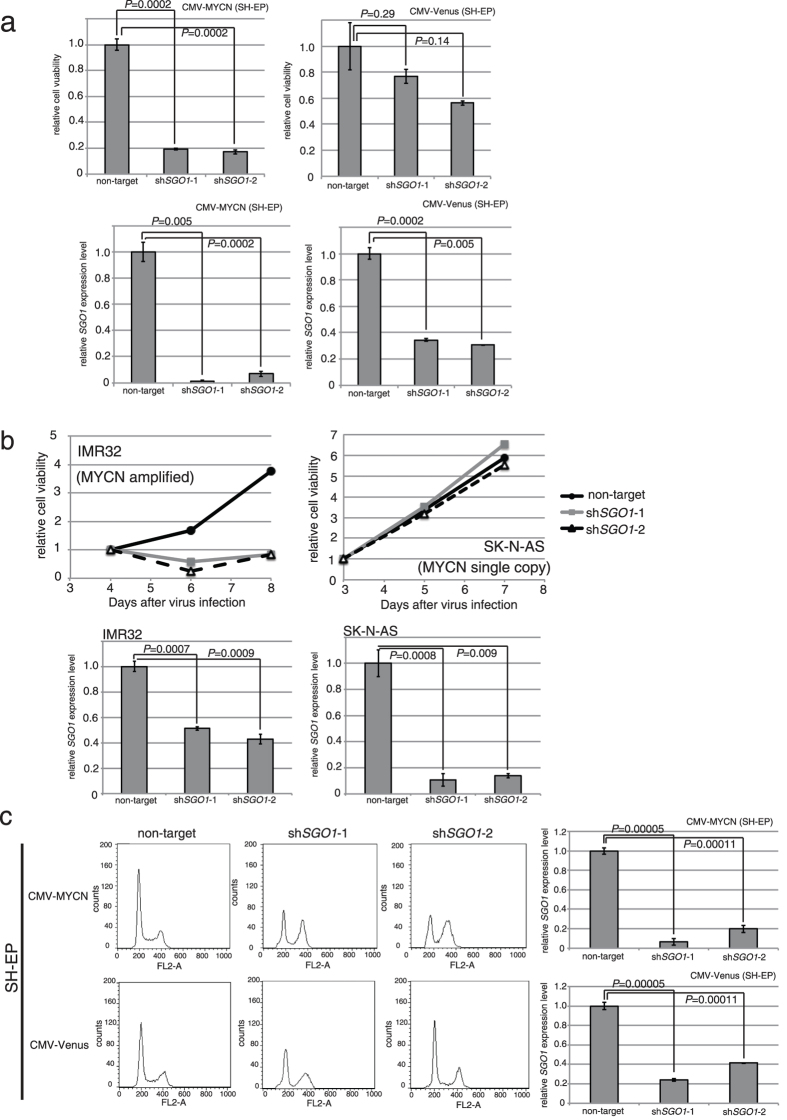
*SGO1* knockdown and MYCN overexpression induce G2/M accumulation. (**a**) *SGO1* knockdown inhibited cell proliferation only in MYCN-overexpressing SH-EP cells. The number of the cells was counted 6 days after non-target or *SGO1*-targeted shRNA lentivirus infection. Upper panel, relative viability; lower panel, SGO1 knockdown efficiency. Data are expressed as the mean ± SE of at least three independent experiments. (**b**) *SGO1* knockdown inhibited cell proliferation only in MYCN-amplified neuroblastoma cell lines. Number of cells counted at 4, 5, 6, 7, or 8 days after non-target or *SGO1*-targeted shRNA lentivirus infection. Upper panel, relative viability; bottom panel, SGO1 knockdown efficiency. (**c**) Flow cytometry profiles of control and SGO1-knockdown cells at 48 hrs after non-target or SGO1-targeted shRNA lentivirus infection. Lower panel shows SGO1 knockdown efficiency.

Next, to determine whether SGO1 affects proliferation of neuroblastoma cells, we knocked down SGO1 in MYCN-overexpressing SH-EP cells using short hairpin RNA (shRNA). SGO1 knockdown severely inhibited the proliferation of MYCN-overexpressing SH-EP cells, but had a much smaller effect in control SH-EP cells (Fig. 3a, upper panel). We confirmed the knockdown efficiency of SGO1 in each cell line (Fig. 3a, bottom panel). We also examined the impact of SGO1 knockdown on several neuroblastoma cell lines and found that SGO1 knockdown severely inhibited the proliferation of MYC-amplified cell line (IMR32). The effect of SGO1 knockdown was much smaller in a cell line harboring a single copy of MYCN (SK-N-AS) (Fig. 3b). Next, we examined cell viability in the presence of caffeine to see whether it inhibited the DDR. Although viability was lower than that in the absence of caffeine, SGO1 knockdown still severely inhibited the proliferation of MYCN-overexpressing SH-EP cells; the effect on control SH-EP cells was much smaller (Fig. S2). These results indicated that overexpression/amplification of MYCN and downregulation of SGO1 exert a synergistic effect on cell proliferation.

We next performed flow cytometry analysis to further investigate the mechanisms underlying inhibition of cell proliferation in SGO1-knockdown cells. A larger fraction of the cell population accumulated in G2/M phase in SGO1-knockdown cells than in control cells transfected with a non-targeting shRNA (Fig. 3c, left panels and Table. S1). The effect of SGO1 knockdown was larger in MYCN-overexpressing cells than in CMV-Venus control cells (Fig. 3c, right panels). Together, these observations indicate that SGO1 is required for efficient progression through G2/M phase in MYCN-overexpressing neuroblastoma cells.

### Downregulation of cohesin induces a growth defect in MYCN-overexpressing cells, but does not induce G2/M arrest

Next, we measured the expression levels of cohesin subunits in *MYCN*-Tg mice (GSE43419). With the exception of *Stag2*, expression of all cohesin subunits increased during neuroblastoma progression (Fig. S3a), as in the case of *Sgo1* (Fig. S1a). To determine whether this expression pattern is also present in humans, we assessed the expression levels of cohesin subunits in neuroblastoma patients using R2. With the exception of *SMC1A*, expression of cohesin subunits was upregulated in *MYCN*-amplified tumors from patients (Fig. S3b). Thus, expression of cohesin subunits is elevated when MYCN levels are high.

To determine whether cohesin plays a role in the growth defect of SGO1-knockdown cells, we knocked down cohesin subunits and monitored the effect on proliferation. Like the SGO1 knockdown, cohesin subunit knockdowns impaired cell proliferation in MYCN-overexpressing cells (Fig. S4a,b), but had no inhibitory effect in control cells (Fig. S4c,d). In contrast to SGO1 knockdown, however, cohesin subunit knockdown did not significantly alter the cell-cycle profile, even in MYCN-overexpressing neuroblastoma cells (Fig. S5a,b). In addition, SGO1 knockdown had no effect on the protein levels of SMC3 in either MYCN-amplified/non-amplified or MYCN-overexpressing/control cells (Fig. S5c). Thus, knockdown of cohesin subunits and MYCN overexpression synergistically inhibit neuroblastoma cell growth, but this effect is not mediated by gross changes in the cell-cycle distribution of the population. Therefore, knockdown of cohesin subunits and SGO1 inhibits growth via different mechanisms; however, further studies are needed to examine this in detail. Based on these findings, we concluded that the phenotype of SGO1-knockdown MYCN-overexpressing cells was distinct from that of cohesin subunit knockdown on the background of MYCN overexpression.

Poly ADP-ribose polymerase (PARP) inhibitors show synergistic responses to cohesin knockdown^37^. To determine whether PARP inhibitors also have inhibitory effects, we examined the viability of SGO1-knockdown cells in the presence or absence of MYCN overexpression. Indeed, SGO1 knockdown and the PARP inhibitor synergistically inhibited proliferation (*P* = 0.007) (Fig. S6). We detected no significant effect of PARP inhibition on the growth of SGO1-knockdown MYCN-overexpressing cells (*P* = 0.36), possibly because the viability of these cells was already quite low.

### SGO1 downregulation induces DNA damage in MYCN-overexpressing neuroblastoma cells

**Figure 4:**
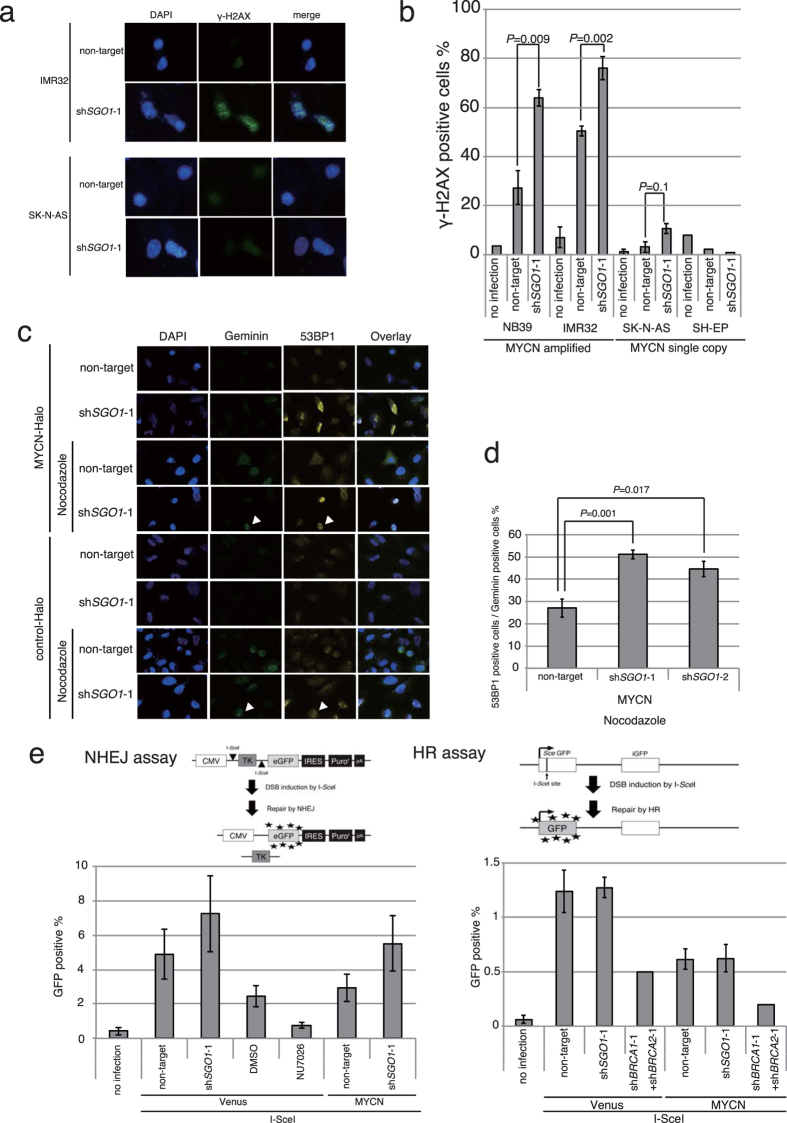
SGO1 knockdown and MYCN overexpression induce DNA damage that is repaired primarily by NHEJ. (**a**) Immunofluorescence of γ-H2AX and DAPI staining of IMR32 (*MYCN*-amplified) and SK-N-AS (*MYCN*-single copy) cells infected with non-targeting or SGO1-specific shRNA. Images were acquired 3 days after infection. (**b**) Percentage of γ-H2AX-positive cells in the cell lines shown in (**a**), as well as in NB39 (*MYCN*-amplified) and SH-EP (*MYCN*-single copy) neuroblastoma cells. Data represent means ± SE of three independent repeats. (**c**) DSB formation in SGO1-knockdown MYCN-overexpressing U2OS cells expressing EYFP-53BP1 and ECFP-Geminin 48 hrs after virus infection. Nocodazole (0.9 μg/ml) was added for 16 hrs to arrest cells in G2/M phase, and cells were counterstained with DAPI. (**d**) Percentage of 53BP1-positive cells relative to the total number of Geminin-positive cells in (**c**). Data are expressed as the mean ± SE of four independent experiments. (**e**) NHEJ efficiency in SGO1-knockdown MYCN-overexpressing H1299dA3-1 #1 cells and HR efficiency in SGO1-knockdown MYCN-overexpressing DR-U2OS cells. NU7026 (a DNA-PK inhibitor) was used as a negative control for the NHEJ assay. shBRCA1 and shBRCA2 were used as a negative control for the HR assay.

To determine whether DNA damage was responsible for the observed impairment in proliferation, we assessed the levels of DNA damage in SGO1-knockdown MYCN-amplified cells. SGO1 knockdown increased the frequency of γ-H2AX foci, which mark sites of DNA damage, in the *MYCN*-amplified cell lines IMR32 and NB39 (Fig. 4a,b). We identified shRNA-infected NB39 cells using a relevant fluorescence (mRFP) probe (Fig. S7). Approximately 30–50% of non-target shRNA-infected NB39 or IMR32 cells displayed γ-H2AX foci (Fig. 4b). The mechanism underlying induction of DNA damage in non-target lentivirus-infected MYCN-amplified cells is unknown. Despite this, the results suggest that knockdown of SGO1 induces additional DNA damage in MYCN-amplified cells (*P* = 0.018 and *P* = 0.002, respectively, in Fig. 4b). We next used U2OS cells expressing ECFP-Geminin and EYFP-53BP1^38^ to determine the cell-cycle phase in which the DNA damage occurred. Geminin is a cell-cycle indicator: its levels are low in G1 phase, gradually increase as the cell cycle progresses, reach a maximum level at mitosis, and rapidly decrease during cytokinesis. Meanwhile, 53BP1 is an indicator of DNA damage that is excluded from irradiation-induced foci during mitosis^39^. As with the frequency of γ-H2AX foci, more 53BP1-positive cells were present in SGO1-knockdown MYCN-overexpressing cells than in SGO1-knockdown control cells (Fig. 4c). Nocodazole treatment led to a significant increase in the number of 53BP1-positive cells among the Geminin-positive SGO1-knockdown MYCN-overexpressing cell population (Fig. 4c,d). As noted above, Geminin and 53BP1 double-positive cells must be in interphase. These results indicated that DNA damage occurred even in interphase in some SGO1-knockdown MYCN-overexpressing cells.

Why did DNA damage occur only in MYCN-overexpressing cells when SGO1 was knocked down? One possibility is that more DNA damage occurred in these cells; alternatively, DNA repair could have been more efficient in control cells. To answer this question, we performed non-homologous end joining (NHEJ)^40^ and HR assays^41^ in cell lines that express GFP when the corresponding type of repair takes place. In the NHEJ assay, SGO1 knockdown tended to increase the proportion of GFP-positive cells in both MYCN-overexpressing and control cell populations (Fig. 4e, NHEJ assay). In the HR assay, the proportion of GFP-positive cells was much lower than in the NHEJ assay (Fig. 4e, HR assay), suggesting that DNA damage induced by SGO1 knockdown is mainly repaired by NHEJ. In addition, the number of GFP-positive cells in the HR assay was higher in control cells than in MYCN-overexpressing cells. Because HR is active primarily in G2 phase, the GFP-positive cells in the HR assay might reflect cells that were damaged in interphase.

Taken together, these data demonstrate that SGO1 knockdown induced DNA damage even in interphase, and that most of the damage was repaired primarily by NHEJ. In MYCN-overexpressing cells, however, persistent SGO1 knockdown-induced damage caused growth arrest. Although the underlying mechanism remains unknown, one possibility is that SGO1 knockdown induces greater DNA damage, which exceeds the capacity for repair.

### Downregulation of SGO1 induces a senescence-like phenotype in MYCN-overexpressing neuroblastoma cells

**Figure 5:**
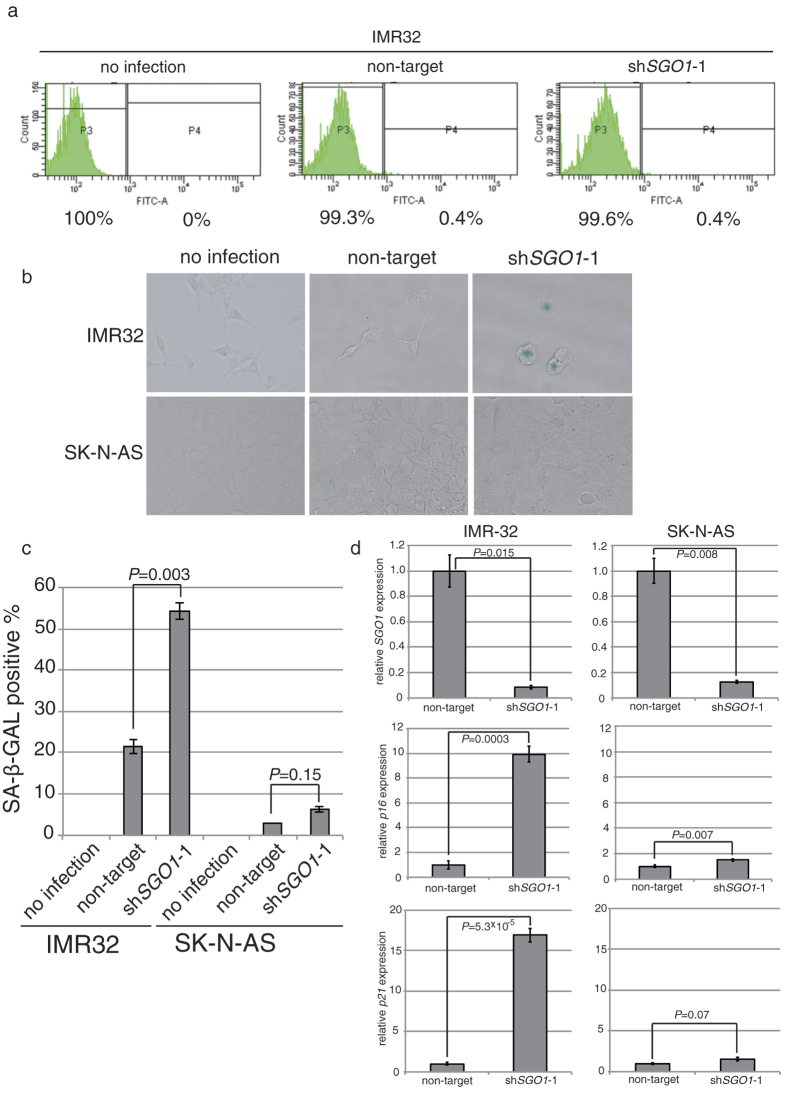
SGO1 knockdown together with MYCN overexpression induces senescence-like phenotype, but not apoptosis. (**a**) TUNEL staining of control or SGO1-knockdown IMR32 cells 6 days after lentivirus infection. (**b**) MYCN-overexpressing or control SH-EP cells infected with lentiviral vectors expressing shRNA against SGO1 were assayed for SA-β-gal activity 6 days after infection. (**c**) The percentage of SA-β-gal-positive cells shown in (**b**). Values represent the mean ± SE of three fields. (**d**) Relative expression of p16 and p21 in the cells shown in (**b**). Values are expressed as the mean ± SE.

We next investigated how SGO1-knockdown MYCN-overexpressing cells halt their proliferation. For this purpose, we first performed a TdT-mediated dUTP nick-end labeling (TUNEL) assay to detect apoptosis in *MYCN*-amplified IMR32 cells. However, TUNEL-positive cells were barely detectable (Fig. 5a). Therefore, we assayed for senescence-associated β-galactosidase (SA-β-GAL), a widely used biomarker of senescence in mammalian cells. Similar to the pattern of γ-H2AX foci (Fig. 4a), the proportion of SA-β-GAL positive cells was greater in IMR32 cells than in the control cell line SK-N-AS (Fig. 5b,c). In addition, expression of p16 and p21 was upregulated in SGO1-knockdown IMR32 cells, but less so in SGO1-knockdown SK-N-AS cells (Fig. 5d). These results demonstrate that SGO1-knockdown MYCN-overexpressing cells stop proliferation by entering senescence-like phenotype rather than undergoing apoptosis.

## Discussion

**Figure 6:**
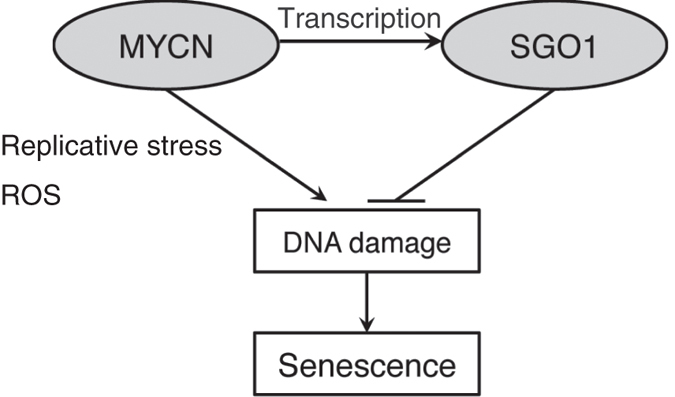
Schematic model of the synergistic phenotype of SGO1 knockdown and MYCN overexpression.

Here, we showed that simultaneous downregulation of SGO1 and upregulation of MYCN induces higher levels of DNA damage, which in turn halts cell proliferation and causes cells to express a senescence-like phenotype (Fig. 6). MYCN induced DNA damage in MYCN-amplified cells, most likely via the production of ROS and replicative stress. On the other hand, since SGO1 is also overexpressed in these cells, the DDR (probably HR) may also be highly activated. As a consequence, cells are viable. In this situation, knockdown of SGO1 impairs the DDR response, resulting in a senescence-like phenotype (Fig. 6). It is still unclear whether this synergistic effect is due to an increased rate of damage or a reduced rate of repair.

The phenotype of SGO1 knockdown varied depending on cell type, potentially due to differences in the expression level of MYC or MYCN. In this regard, SGO1 knockdown alone induces cellular senescence in human fibroblasts^42^, possibly because growing fibroblasts express a high level of MYC^43^. Subsequently, SGO1 knockdown halts the proliferation of HCT116, NB-4, EOL-1, and U937 cell lines^5^,^9^, all of which express MYC. Together, these observations support the notion of a strong genetic interaction between MYC and SGO1.

SGO1 knockdown induced DNA damage in MYCN-overexpressing neuroblastoma cells (Fig. 4a,b), resulting in G2/M accumulation (Fig. 3b). This damage was repaired by NHEJ to a greater extent than by HR (Fig. 4d). The higher level of DNA damage in SGO1-knockdown cells suggests that SGO1 positively regulates the HR pathway. If HR were fully functional in SGO1-knockdown cells, the number of GFP-positive cells detected in the HR assay would be comparable to that observed in the NHEJ assay. Together, these observations suggest that DNA damage induced by SGO1 downregulation is not fully repaired in G2/M phase by HR, resulting in accumulation at G2/M.

In SGO1-knockdown MYCN-overexpressing cells, some DNA damage occurred in interphase (Fig. 4c). Several mechanisms might be responsible for induction of DSBs in interphase. First, as demonstrated previously in HeLa cells, SGO1 protects cohesin on chromosomal arms during interphase via the HP1-SGO1 pathway^44^. When SGO1 is knocked down, cohesin is released from both the chromosomal arm and pericentromeric regions even in interphase, potentially resulting in DNA damage. Second, SGO1 is involved in the DNA repair system, and as noted above may positively regulate HR. Moreover, SGO1 interacts with Ku70^45^, a subunit of DNA-PK holoenzyme, which is essential for recognition of DSBs in NHEJ. The Ku complex (Ku70–Ku80), the DNA damage sensor for NHEJ, also functions as a DNA damage sensor for HR in the absence of the Mre11-Rad50-Nbs1 (MRN) complex, the usual damage sensor for HR^46^, suggesting that the Ku complex plays a role in DNA repair beyond NHEJ. Third, SGO1 may regulate the expression of DDR proteins; if so, SGO1 knockdown would decrease the abundance of DDR factors and compromise the efficiency of repair, thereby increasing the frequency of DSBs. Consistent with this hypothesis, SGO1 interacts with condensin in budding yeast^47^, and SMC2, a subunit of condensin, regulates transcription of DDR genes in cooperation with MYCN^35^.

Finally, we showed that, in addition to being a target of YAP/TAZ/TEAD (the Hippo pathway) and AP-1, SGO1 is also a target of MYCN (Fig. 2). Like many other genes involved in replication and cell-cycle control, *MYC* itself is also a target of YAP/TAZ^4^, suggesting that both MYCN or MYC and SGO1 can amplify signals from the Hippo pathway. Therefore, we propose that SGO1 is a potential molecular target for the treatment of *MYCN*-amplified neuroblastoma and tumors bearing YAP/TAZ hyperactivation.

## Methods

### Mice

MYCN Tg mice^48^ and wild type mice were maintained in the animal facility at Nagoya University Graduate School of Medicine, where they were housed in a controlled environment and provided with standard nourishment and water. Normal ganglia and precancerous and tumor tissues from wild type, hemizygous, and homozygous MYCN Tg mice were dissected and minced before total RNA was extracted. All methods were performed in accordance with the relevant guidelines and regulationswas approved by the Animal Care and Use Committee of Nagoya University Graduate School of Medicine, Nagoya, Japan.

### Gene expression array data

Previously published data^35^ were used for the expression analyses. These data were deposited in the Gene Expression Omnibus database of NCBI (http://www.ncbi.nlm.nih.gov/geo) under accession number GSE43419.

### Cell lines and lentiviruses

Cell lines used in this study are listed in Supplementary Table S2. Cells were cultured as previously described^35^,^40^,^38^,^41^. For the knockdown experiments, replication-defective and self-inactivating lentiviruses were prepared as previously described^35^. We used a 2^nd^ generation packaging system based on psPAX2 and pMD2.G vectors.

### Plasmids and primers

Commercially available non-targeting (Sigma) and SGO1- or cohesin subunit-specific shRNA vectors (Thermo Scientific Open Biosystems) were used. The shRNA sequences are listed in Table S3. The primers used in this study are listed in Supplementary Table S4.

### Isolation of RNA, RT-PCR, and qPCR

Total RNA was isolated from cells using the RNeasy Plus Mini kit (Qiagen) or the ISOGEN II reagent (Nippongene). cDNA was prepared using ReverTra Ace qPCR RT kit with gDNA Remover (TOYOBO). Quantitative PCR analyses were performed using the KAPA SYBR Fast qPCR kit (KAPA Biosystems) on an Applied Biosystems 7500 Real-Time PCR System. Relative gene expression levels were determined using the ΔΔC_T_ method.

### Protein preparation and immunoblotting

Whole-cell lysates were prepared using Cell Lytic M solution (C2978, Sigma) containing a protease inhibitor cocktail (11697498001, Roche) and subjected to SDS-PAGE. Immunoblotting was performed as previously described^35^. Western blots were analyzed using ImageJ software.

### Antibodies

The following antibodies were used: anti-SGO1 mouse monoclonal (ab58023, Abcam); anti-SGO1 rabbit polyclonal (GTX117103, GeneTex, and ab21633, Abcam); anti-MYCN mouse monoclonals OP13 (Calbiochem), NB200-109 (Novus Biologicals), and sc-53993 (Santa Cruz Biotechnology); anti-MYCN rabbit polyclonal (#9405, Cell Signaling Technology); anti-phospho-histone H2A.X (Ser139) mouse monoclonal (JBW301, Millipore); anti-SMC3 rabbit polyclonal (A300-060A, Bethyl laboratories); anti-β-ACTIN (8H10D10) mouse monoclonal (#3700, Cell Signaling Technology); normal mouse IgG (#12–371, Millipore); goat anti-mouse IgG (H+L) secondary antibody, Alexa Fluor^®^ 488 conjugate (A-11001, Thermo Fisher Scientific); anti-mouse IgG, HRP-linked (#7076S, Cell Signaling Technology); and anti-rabbit IgG, HRP-linked (#7074S, Cell Signaling Technology) antibodies.

### ChIP

Cultured sub-confluent SH-EP cells expressing Venus or MYCN were treated with 1% (v/v) formaldehyde for 10 min at room temperature. Cross-linking was stopped by addition of glycine to a final concentration of 125 mM. The cells were washed with cold PBS and then harvested. The cells were pelleted, frozen at −70 °C, and then lysed by mechanical disruption. The Halo-ChIP system (Promega) was then used according to the manufacturer’s instructions, with a slight modification: an anti-MYCN antibody and Dynabeads-protein G (Dynal) were used. DNA was purified twice using phenol-CIAA and then PCR-amplified using primers spanning the MYCN-binding site upstream of the *SGO1* gene. Cells immunoprecipitated with control IgGs were used as a negative control.

### Cell viability

MYCN-overexpressing or control Venus-expressing SH-EP cells were infected with non-target or shSGO1 lentivirus. 4, 5, 6, 7, or 8 days later, the cells were counted using Cell Counting Kit-8, according to the manufacturer’s protocol (Dojindo Laboratories).

### FACS

DNA content was determined on a FACSCalibur instrument (BD Bioscience), and data were analyzed using Cell Quest software.

### Immunofluorescence analysis

Cells were grown on glass coverslips in 4 well plates and then fixed with 4% paraformaldehyde for 1 hr at room temperature. Fixed cells were permeabilized with PBS containing 1% NP-40 for 10 min at room temperature and incubated with an anti-γ-H2AX antibody (1:1000 dilution) for 1 hr at room temperature, followed by an Alexa Fluor 488-conjugated anti-mouse IgG (Molecular Probes) (1:1000 dilution) for 30 min at room temperature. After incubation with 4′6-diamidino-2-phenylindole (0.1 μg/ml) for 5 min, cells were mounted in FluorSave Reagent (Millipore). All images were obtained using BZ-9000 (Keyence) or LSM710 (Carl-Zeiss) and subsequently processed using MetaMorph or Zen software and Adobe Photoshop.

### NHEJ assay

The NHEJ assay was performed as described^40^. Briefly, H1299dA3-1#1 cells^40^ were cultured in 24 well plates, infected with shRNAs, and transfected with the I-SceI expression plasmid (pCBASce). After 72 hrs, cells were harvested and single cell suspensions were analyzed in a FACS Aria III cytometer (Becton Dickinson). NU7026 (35 μM; a DNA-PK inhibitor) treatment was used as a negative control.

### HR assay

The HR assay was performed as previously described^41^. Briefly, DR-U2OS cells^41^ were cultured in 24 well plates and infected with shRNAs and transfected with I-SceI expression plasmid (pCBASce). After 72 hrs, cells were harvested and single cell suspensions were analyzed using a FACS Aria III cytometer (Becton Dickinson).

### Image analysis

Cells were infected with lentivirus to introduce sh*SGO1* and induce MYCN overexpression. After 24 hrs, nocodazole (0.9 μg/ml) was added to arrest cells at G2/M. After another 24 hrs, cells were counterstained with DAPI. Cells were then examined under an LSM710 microscope (Carl-Zeiss), and images were analyzed using Image Zen software (Zeiss).

### TUNEL assay

The TUNEL assay was performed using the APO-DIRECT kit (BD Pharmingen), and the cells were analyzed on a FACSCanto flow cytometer (BD Pharmingen) using the Diva software.

### β-galactosidase assay

SA-β-GAL assays were performed using the Senescence Detection kit (K320-250, Biovision).

### Statistical analysis

Results are expressed as the mean ± SE. Homoscedasticities were checked using the *f* test. Statistical significance was evaluated with a 2-tailed, unpaired *t* test and significance set at P < 0.05.

## Additional Information

**How to cite this article**: Murakami-Tonami, Y. *et al*. SGO1 is involved in the DNA damage response in MYCN-amplified neuroblastoma cells. *Sci. Rep.* **6**, 31615; doi: 10.1038/srep31615 (2016).

## Supplementary Material

Supplementary Information
