# Supplementary material for: SGO1 is involved in the DNA damage response in MYCN-amplified neuroblastoma cells
Source: Sci Rep. 2016 Aug 19;6:31615. doi: 10.1038/srep31615 (PMC4990925; doi:10.1038/srep31615)
Supplement: Supplementary Information [file srep31615-s1.pdf]

Supplementary materials

Title:

# SGO1 is involved in the DNA damage response in MYCN- amplified neuroblastoma cells

Authors:

Yuko Murakami-Tonami, Haruna Ikeda, Ryota Yamagishi, Mao Inayoshi, Shiho Inagaki, Satoshi Kishida, Yosuke Komata, Jan Koster, Ichiro Takeuchi, Yutaka Kondo, Tohru Maeda, Yoshitaka Sekido, Hiroshi Murakami and Kenji Kadomatsu

**Supplementary figure legends**

**Figure S1.** Expression of *SGO1* and *SGO2* correlates with expression of *MYCN* or *MYC* in various cancers.

(a) Heatmap of *Sgo*1 gene expression in ganglia from wild-type mice (lanes 1 and 2), precancerous (lanes 3 and 4) and tumor lesions (lanes 5 and 6) from homozygous *MYCN*-Tg mice, and tumor lesions (lanes 7 and 8) from hemizygous *MYCN*-Tg mice (GSE43419).

(b) *SGO1* expression in patients with *MYCN*-amplified or non-amplified tumors, based on datasets from the R2 bioinformatics platform (http://r2.amc.nl).

(c) *SGO1* expression correlates with *MYCN* or *MYC* expression in various cancers. Data were generated using the TCGA pan-cancer gene expression database (data version 2013-12-18). Red columns indicate high *SGO1* or *SGO2* expression with high *MYCN* expression; blue columns indicate high *SGO1* or *SGO2* expression with high *MYC* expression.

**Figure S2.** *SGO1* knockdown inhibits cell proliferation only in MYCN-overexpressing SH-EP cells, even in the presence of caffeine.

The number of the cells was counted 6 days after non-target or *SGO1*-targeted shRNA lentivirus infection. Caffeine (2 mM) was added 24 hrs after virus infection. Upper panel, relative viability; lower panel, SGO1 knockdown efficiency. Data were expressed as the mean ± SE of at least three independent experiments.

**Figure S3.** Expression of cohesin subunits in mouse and human neuroblastoma cells.

(a) Expression profiles of cohesin complex subunits in ganglia of wild-type mice and in precancerous and tumor lesions from homozygous *MYCN*-Tg mice (GSE43419).

(b) *SMC1A*, *SMC3*, *RAD21*, and *STAG2* expression in patients with *MYCN*-amplified or non-amplified tumors, using datasets from the R2 bioinformatics platform (http://r2.amc.nl).

**Figure S4.** Cell growth after cohesin subunit knockdown is dependent on *MYCN* overexpression.

(a) Effects of MYCN overexpression and cohesin subunit knockdown on the viability and proliferation of SH-EP (*MYCN*-single copy) cells. Number of cells 6 days after lentivirus infection of non-target or cohesin subunit-targeted shRNA.

(b) mRNA level of each cohesin subunit in the cells shown in (a).

(c) Effects of cohesin subunit knockdown on the viability and proliferation of SH-EP (*MYCN*-single copy) cells.

(d) mRNA level of each cohesin subunit in the cells shown in (c).

**Figure S5.** The majority of cohesin subunit-knockdown cells are in G1 phase.

(a) FACS analysis of MYCN-overexpressing and/or cohesion subunit-knockdown SH-EP cells. Samples were harvested 2 days after lentivirus infection of non-target or cohesin subunit-targeted shRNA.

(b) mRNA levels of each cohesin subunit in the knockdown cells shown in (a).

(c) SMC3 protein levels in neuroblastoma cells did not change much when SGO1 was knocked down. Samples were harvested 3 days after lentivirus infection with non-target or *SGO1*-targeted shRNA.

**Figure S6.** A PARP inhibitor acts synergistically with *SGO1* knockdown.

The effect of MC2050 (a PARP inhibitor) on MYCN-overexpressing and/or SGO1-knockdown SH-EP cells.

**Figure S7.** Almost all cells were infected by a lentivirus containing non-target or SGO1-targeted shRNA. To identify shRNA-infected cells, we performed an experiment similar to that depicted in Fig. 4b, in addition to infecting NB39 cells with a relevant fluorescence probe (mRFP).

# Figure S1

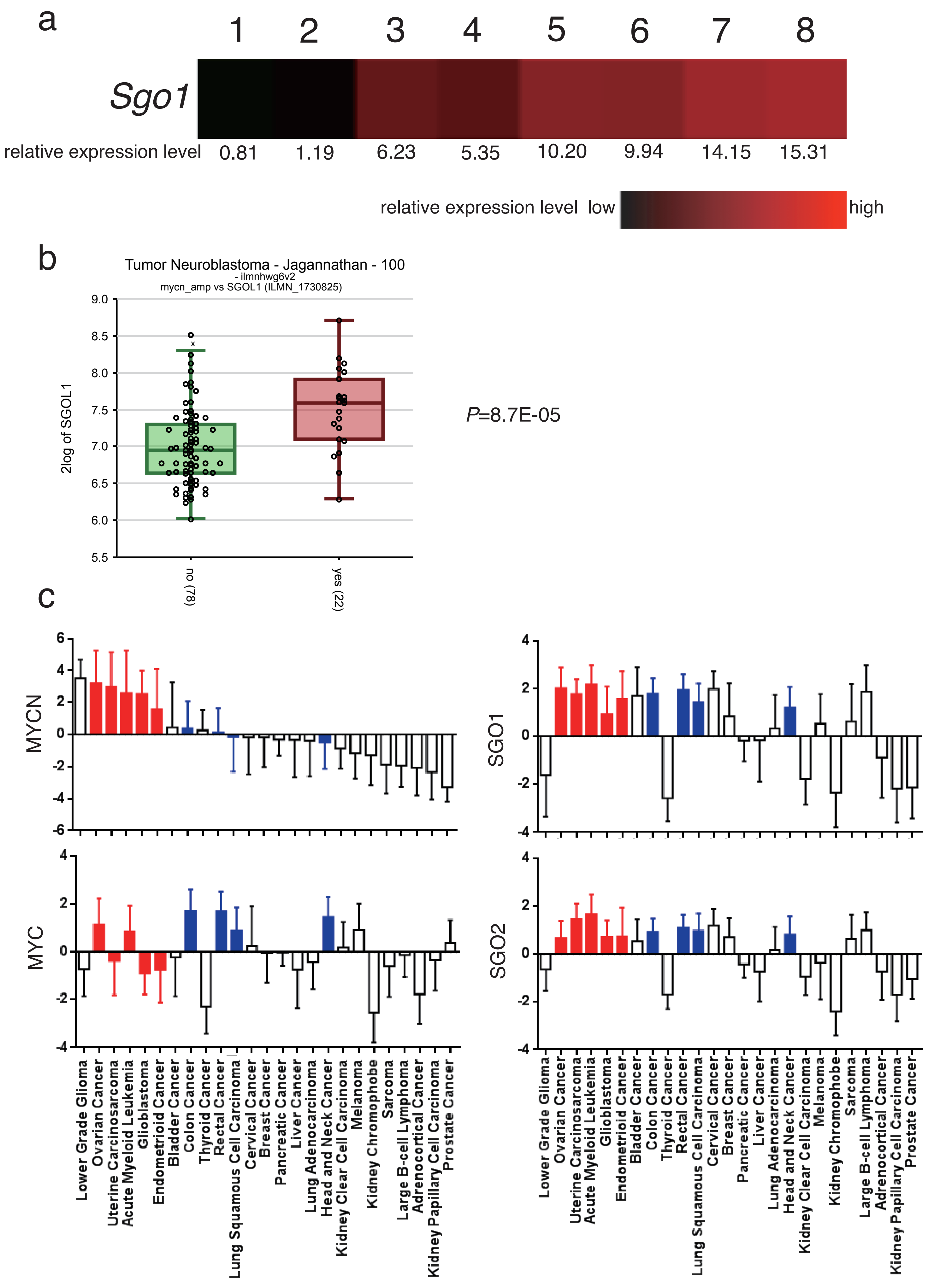
a
1 2 3 4 5 6 7 8
Sgo1
relative expression level 0.81 1.19 6.23 5.35 10.20 9.94 14.15 15.31
relative expression level low high
b
Tumor Neuroblastoma - Jagannathan - 100
- ilmnhwg6v2
mycn_amp vs SGOL1 (ILMN_1730825)
2log of SGOL1
9.0 8.5 8.0 7.5 7.0 6.5 6.0 5.5
no (78)
yes (22)
P=8.7E-05
c
MYCN
SGO1
MYC
SGO2
Lower Grade Glioma
Ovarian Cancer
Uterine Carcinosarcoma
Acute Myeloid Leukemia
Glioblastoma
Endometrioid Cancer
Bladder Cancer
Colon Cancer
Thyroid Cancer
Rectal Cancer
Lung Squamous Cell Carcinoma
Cervical Cancer
Breast Cancer
Pancreatic Cancer
Liver Cancer
Lung Adenocarcinoma
Head and Neck Cancer
Kidney Clear Cell Carcinoma
Melanoma
Kidney Chromophobe
Sarcoma
Large B-cell Lymphoma
Adrenocortical Cancer
Kidney Papillary Cell Carcinoma
Prostate Cancer

# Figure S2

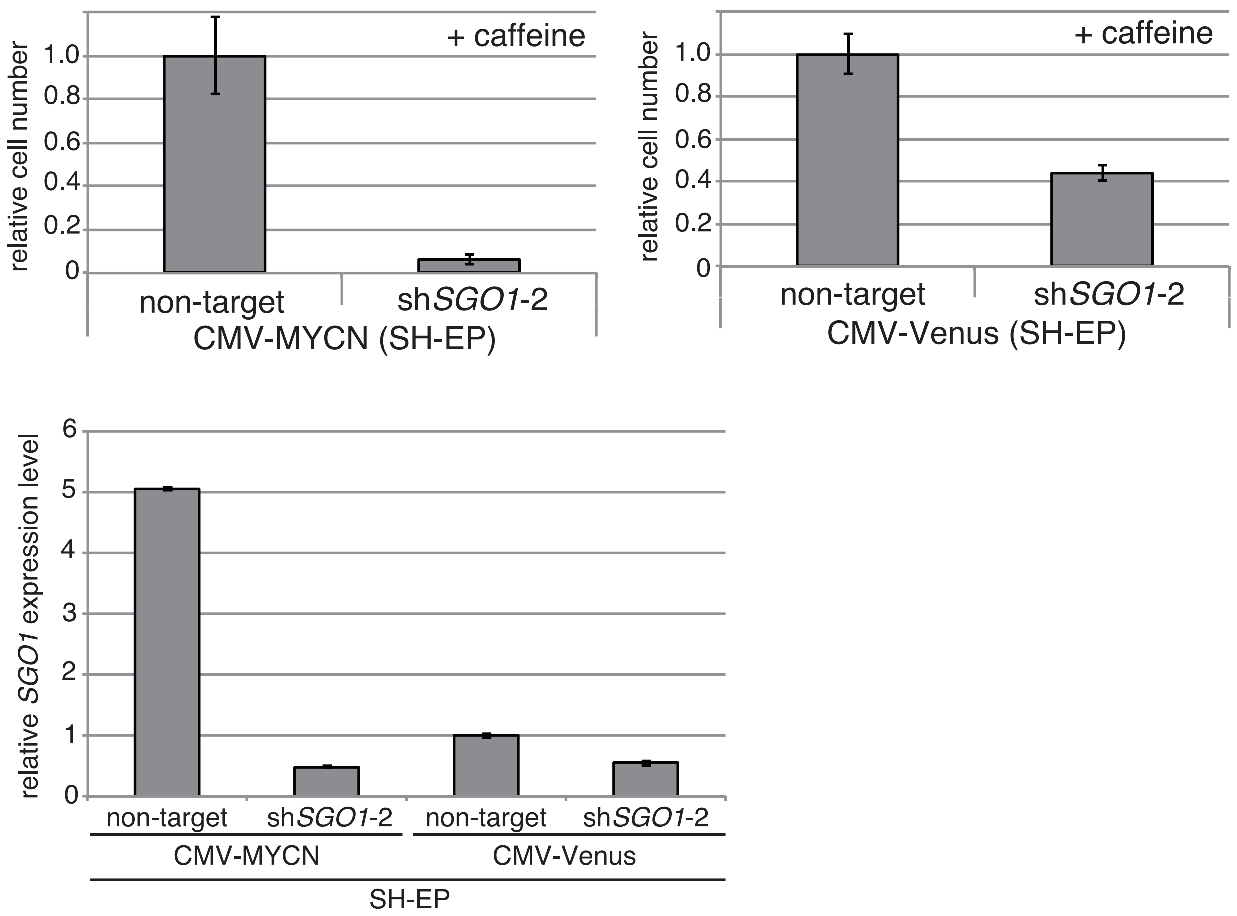

+ caffeine
relative cell number
1.0
0.8
0.6
0.4
0.2
0
non-target
shSGO1-2
CMV-MYCN (SH-EP)
+ caffeine
relative cell number
1.0
0.8
0.6
0.4
0.2
0
non-target
shSGO1-2
CMV-Venus (SH-EP)
relative SGO1 expression level
6
5
4
3
2
1
0
non-target
shSGO1-2
non-target
shSGO1-2
CMV-MYCN
CMV-Venus
SH-EP

# Figure S3

a

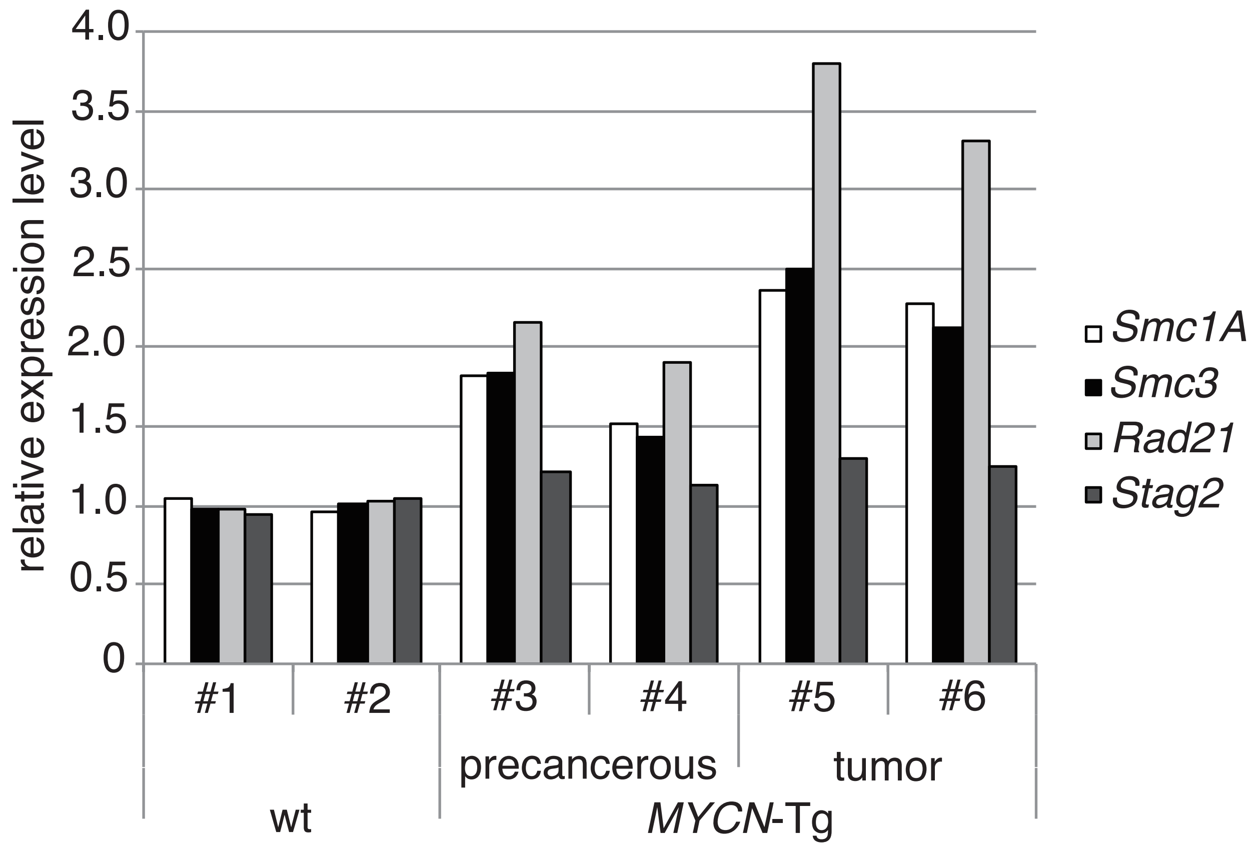

relative expression level
4.0
3.5
3.0
2.5
2.0
1.5
1.0
0.5
0
#1
#2
#3
#4
#5
#6
precancerous
tumor
wt
MYCN-Tg
Smc1A
Smc3
Rad21
Stag2


b

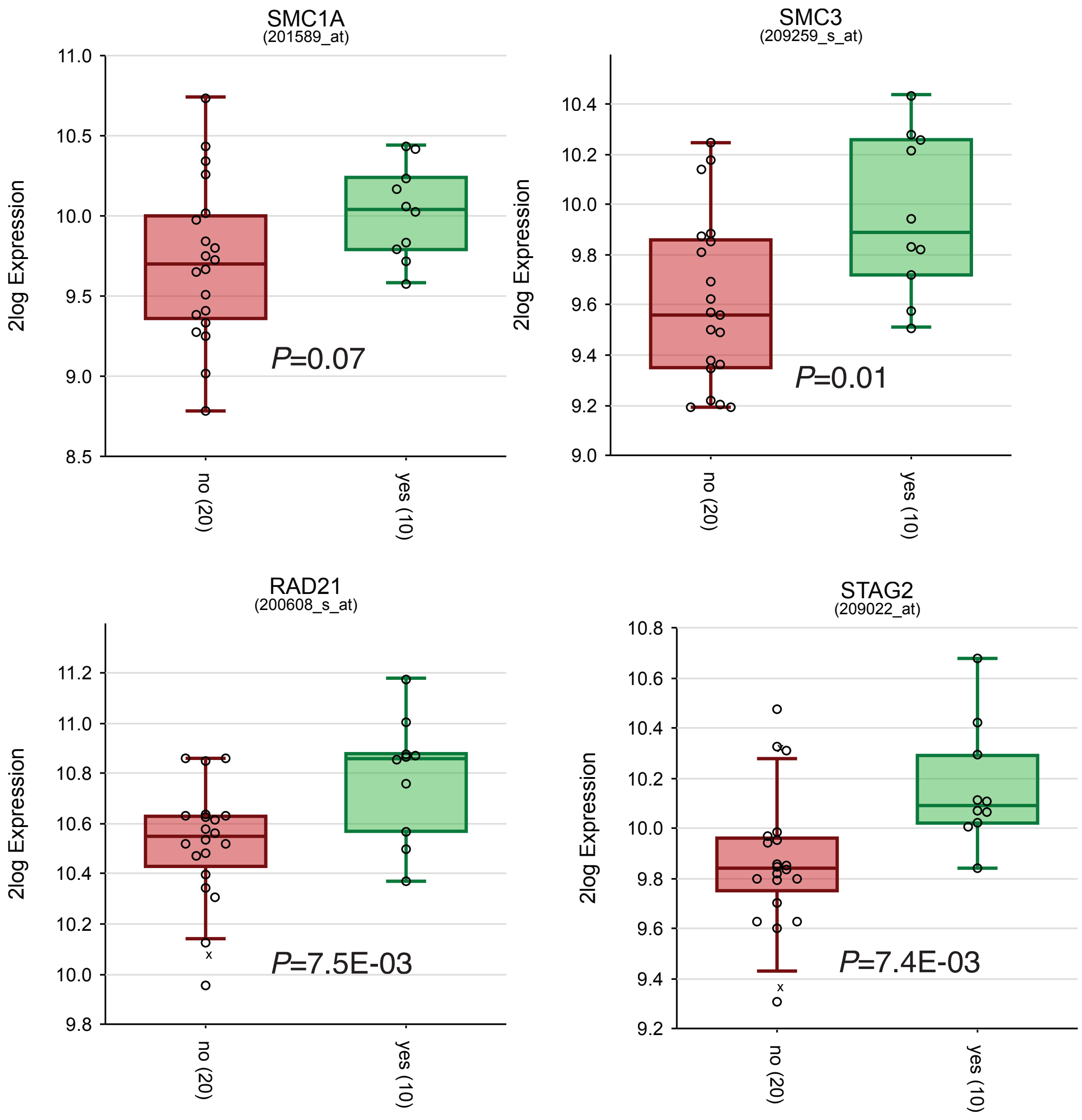

SMC1A
(201589_at)
2log Expression
P=0.07
no (20)
yes (10)
SMC3
(209259_s_at)
2log Expression
P=0.01
no (20)
yes (10)
RAD21
(200608_s_at)
2log Expression
P=7.5E-03
no (20)
yes (10)
STAG2
(209022_at)
2log Expression
P=7.4E-03
no (20)
yes (10)

# Figure S4

a

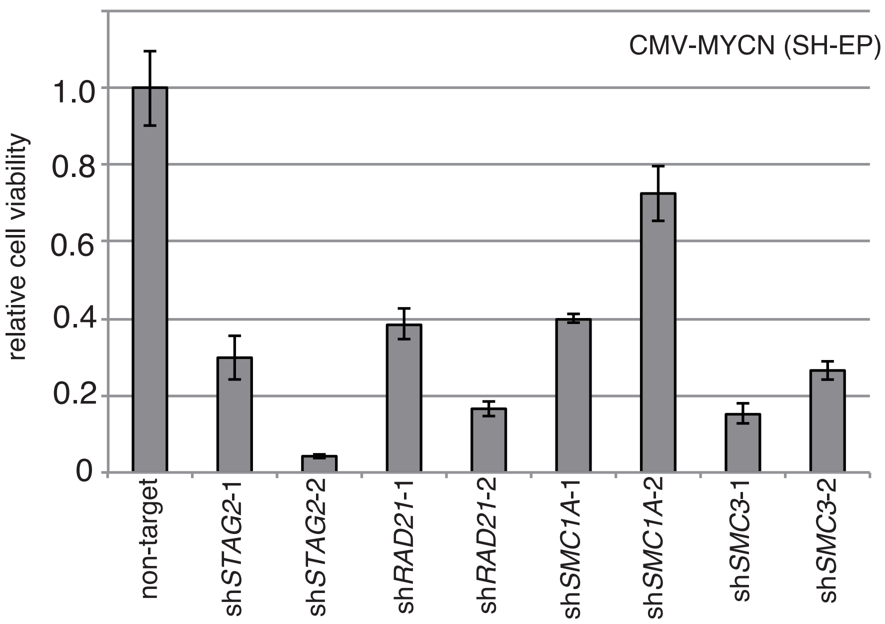
CMV-MYCN (SH-EP)
relative cell viability
1.0
0.8
0.6
0.4
0.2
0
non-target
shSTAG2-1
shSTAG2-2
shRAD21-1
shRAD21-2
shSMC1A-1
shSMC1A-2
shSMC3-1
shSMC3-2

b

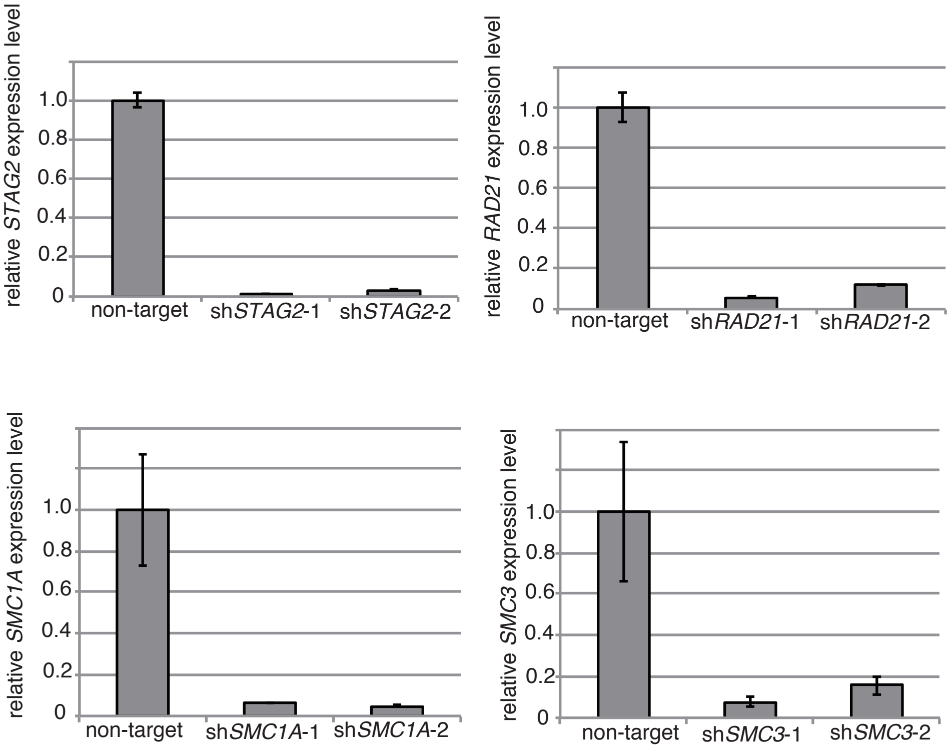
relative STAG2 expression level
non-target
shSTAG2-1
shSTAG2-2
relative RAD21 expression level
non-target
shRAD21-1
shRAD21-2
relative SMC1A expression level
non-target
shSMC1A-1
shSMC1A-2
relative SMC3 expression level
non-target
shSMC3-1
shSMC3-2
1.0
0.8
0.6
0.4
0.2
0

Figure S4 (Continued)

c

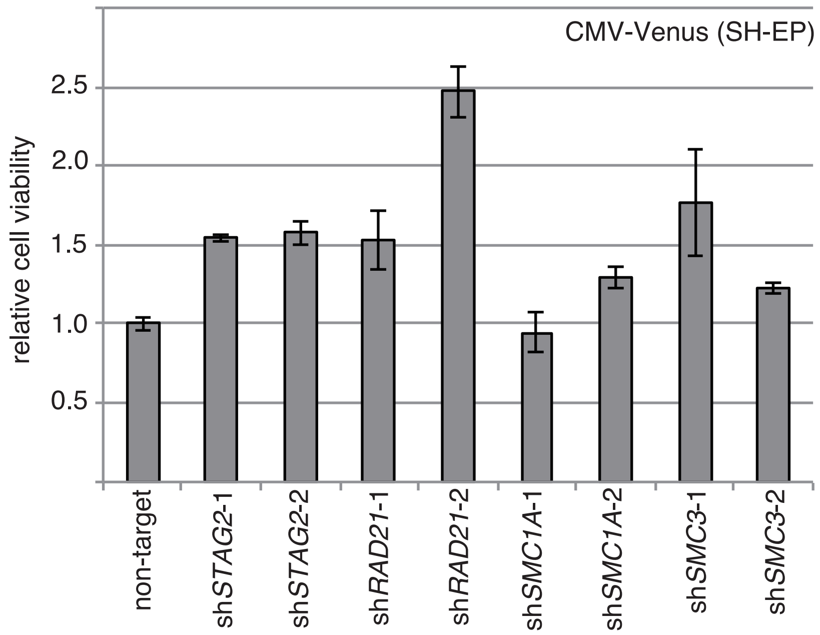


d

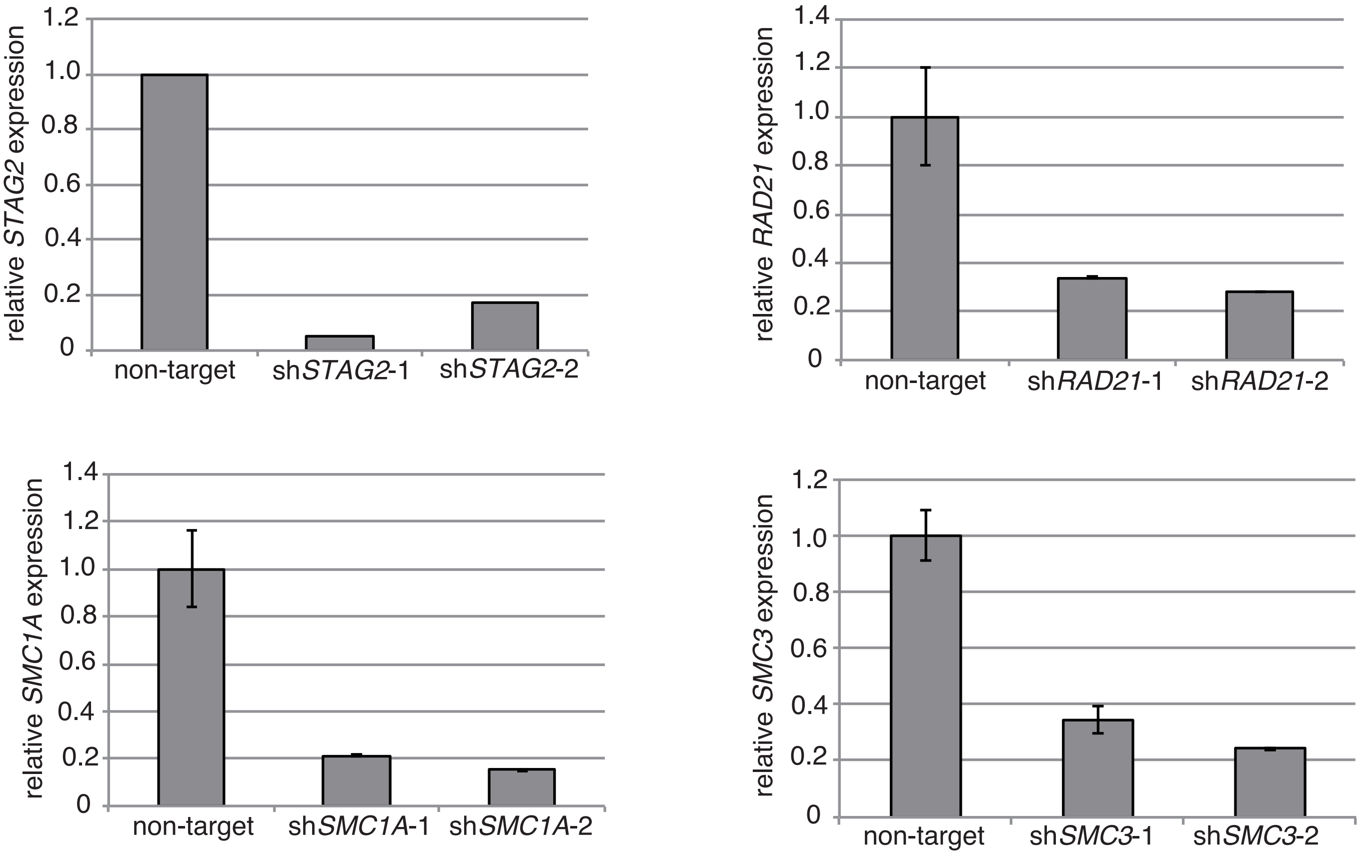

# FigureS5

a

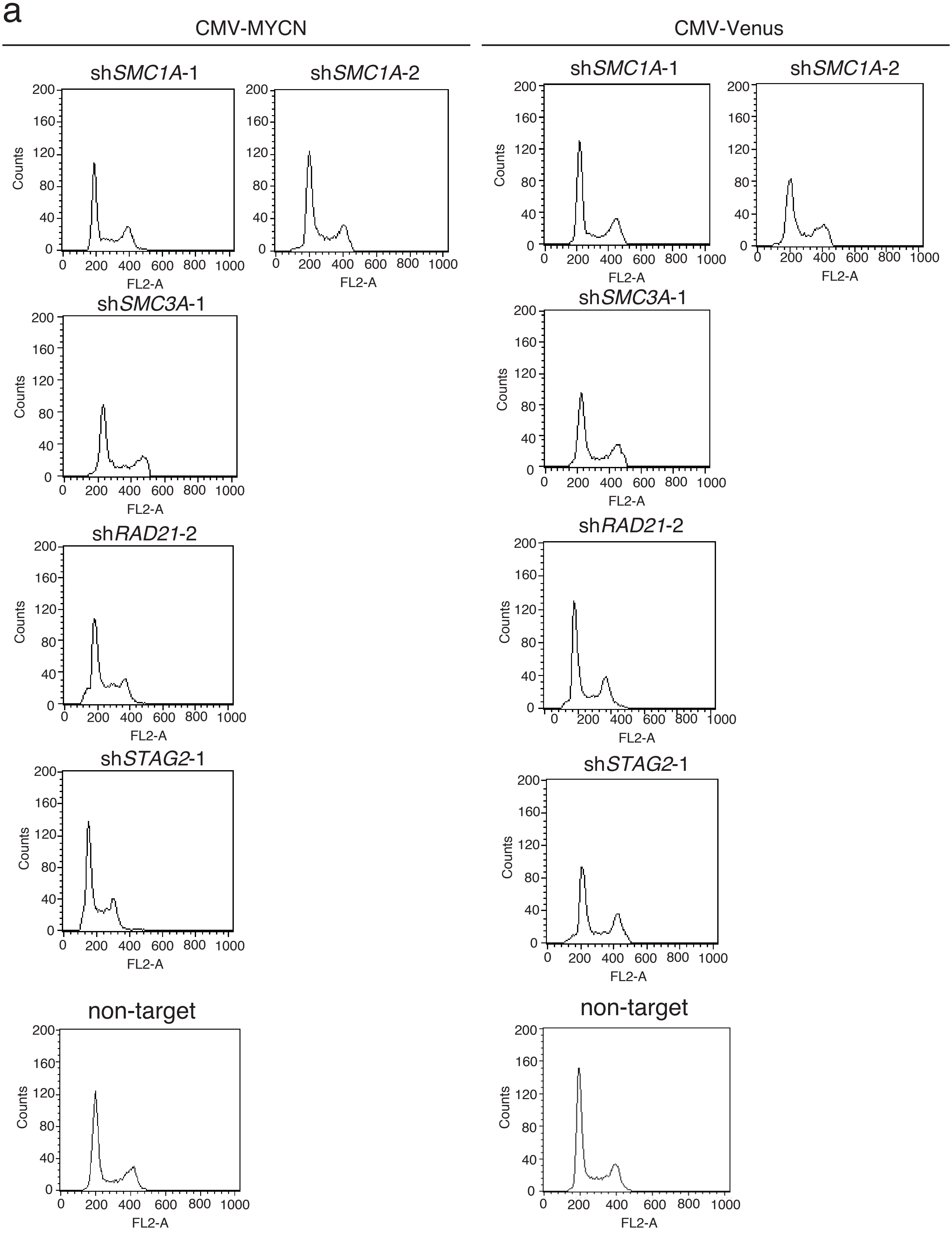

CMV-MYCN
CMV-Venus
shSMC1A-1
shSMC1A-2
shSMC3A-1
shRAD21-2
shSTAG2-1
non-target
Counts
FL2-A
0
40
80
120
160
200
0
200
400
600
800
1000

# Figure S5 (Continued)

b

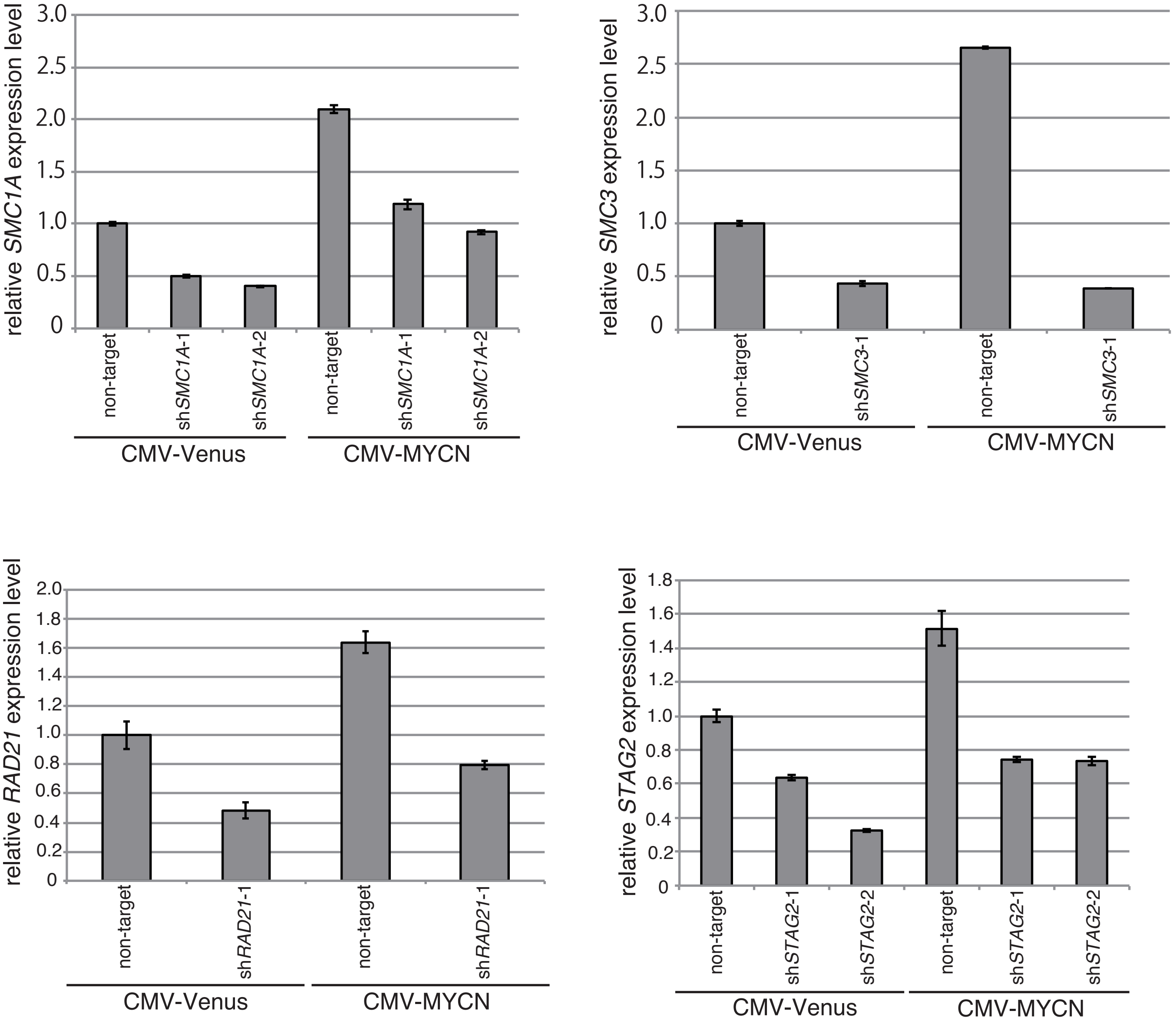


c

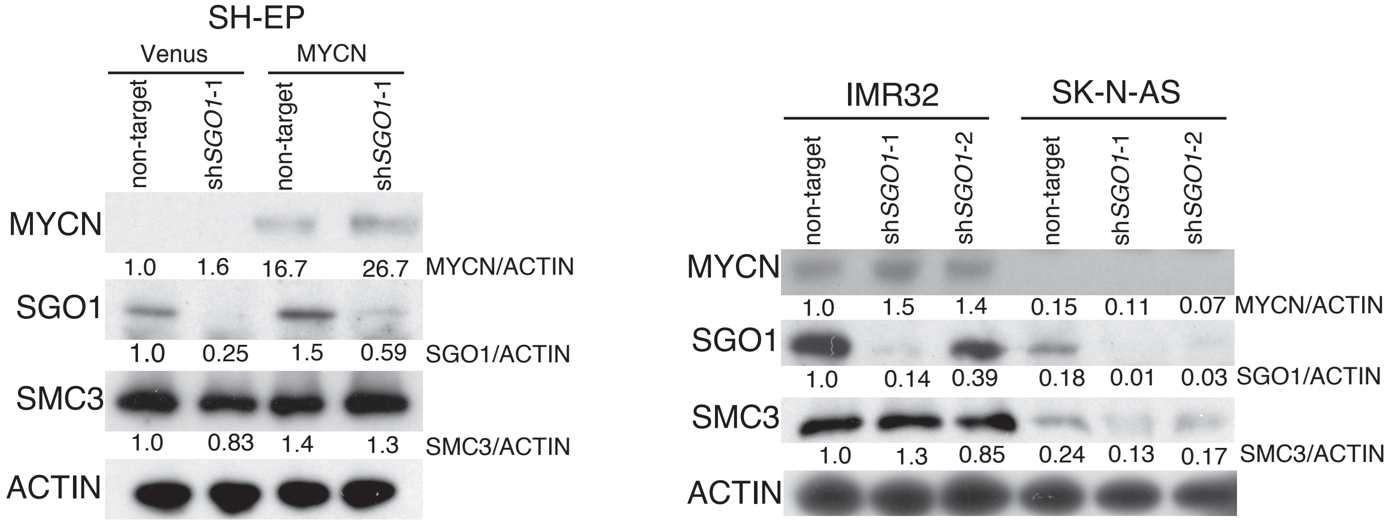

# Figure S6

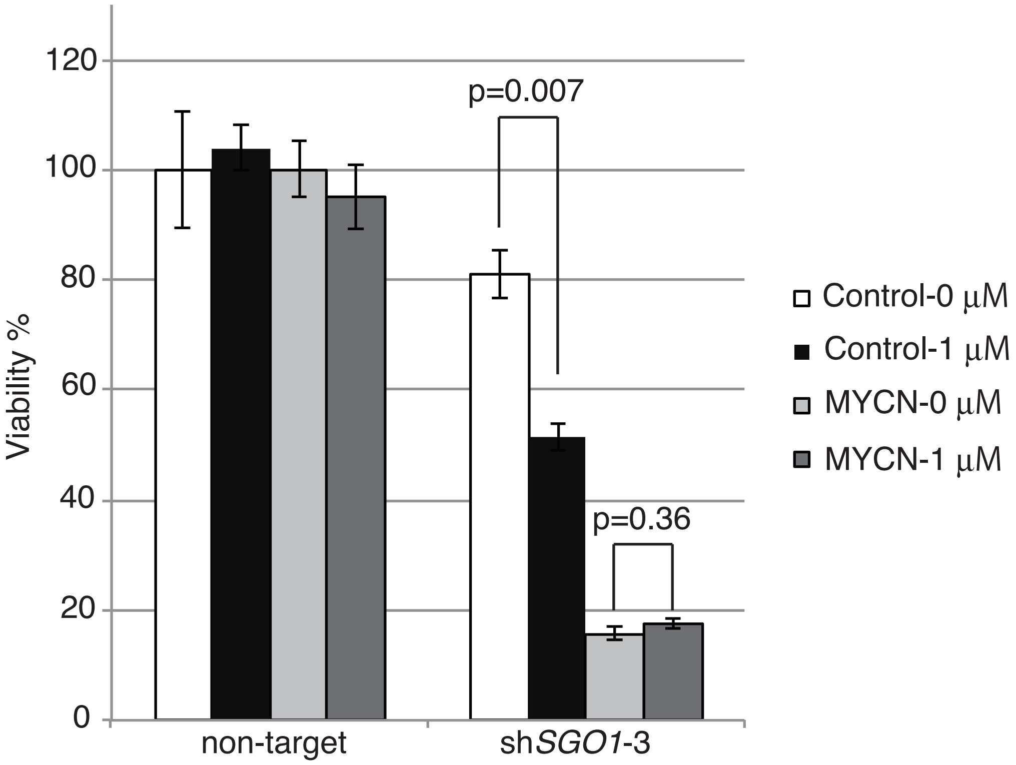

120
100
80
60
40
20
0
Viability %
p=0.007
p=0.36
non-target
shSGO1-3
Control-0 µM
Control-1 µM
MYCN-0 µM
MYCN-1 µM

# Figure S7

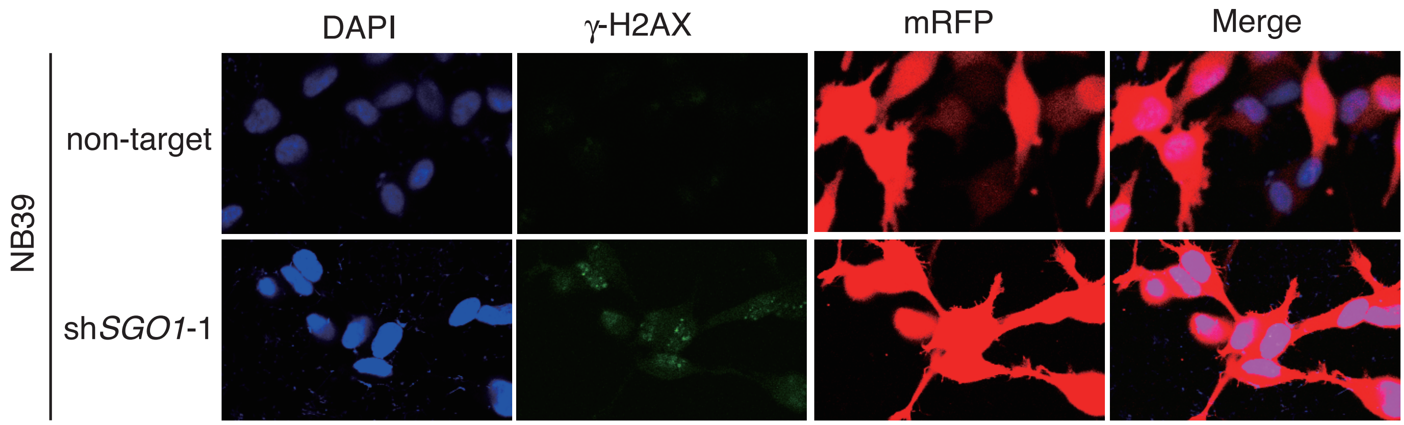

The cell number and %cells of cell cycle

Supplemental Table 1

Cell numbers and % cells of cell cycle

| | | | | % cells | | |
|---|---|---|---|---|---|---|
| | | | cell count | G1 | S | G2/M |
| SH-EP | CMV-MYCN | non-target | 8651 | 50.7 | 31.5 | 17.8 |
| | | sh*SGO1*-1 | 6144 | 31.1 | 22.2 | 46.8 |
| | | sh*SGO1*-2 | 7302 | 31.1 | 15.5 | 53.5 |
| | CMV-Venus | non-target | 7389 | 59.5 | 27 | 13.5 |
| | | sh*SGO1*-1 | 5889 | 59.4 | 14.4 | 26.2 |
| | | sh*SGO1*-2 | 6523 | 54 | 15.5 | 30.5 |

Supplemental Table 2

Cell lines

| | | |
|---|---|---|
| SK-N-BE | Neuroblastoma | ATCC (CRL-2271) |
| IMR32 | Neuroblastoma | JCRB (JCRB9050) |
| NB39 | Neuroblastoma | Gift from Dr. Chiba |
| SK-N-AS | Neuroblastoma | ATCC (CRL-2137) |
| SH-EP | Neuroblastoma | Gift from Dr. Schwab |
| SH-EP (Venus) | Neuroblastoma | Murakami-Tonami et al. Cell Cycle (2014) |
| SH-EP (MYCN) | Neuroblastoma | Murakami-Tonami et al. Cell Cycle (2014) |
| H1299dA3-1 #1 | Lung Cancer | Gift from Dr. Kohno (Ogiwara et al Oncogene (2011)) |
| DR-U2OS | Osteosarcoma | Gift from Dr. Jasin (Xia et al Mol Cell (2006)) |
| U2OS cells expressing mCherry-Rad52, EYFP-53BP1, ECFP-Geminin | Osteosarcoma | Gift from Dr. Lahav (Karanam et al Mol Cell (2012)) |

Supplemental Table 3

Plasmids

| Name | No. | sequence | |
|---|---|---|---|
| shSGO1-1 | TRCN0000074150 | TTCTTCAAGAGGAATTTGCGG | GE Dharmacon |
| shSGO1-2 | TRCN0000074151 | ATCCTTCAGGCTAAGATGAGG | GE Dharmacon |
| shSGO1-3 | TRCN0000074152 | TACGAACAGATACAGTTCTAG | GE Dharmacon |
| shSMC1A-1 | TRCN0000062553 | ATTGATTTCATCAATACGCCG | GE Dharmacon |
| shSMC1A-2 | TRCN0000062555 | TATACTGAATACAGTCCCGGC | GE Dharmacon |
| shSMC3-1 | TRCN0000160156 | ATTGAGATAAGTCTCTACTCG | GE Dharmacon |
| shSMC3-2 | TRCN0000160366 | AACTTCTGGAATACTTCACTG | GE Dharmacon |
| shRAD21-1 | TRCN0000148110 | TTCTTCAGGTAAAGTAATGGC | GE Dharmacon |
| shRAD21-2 | TRCN0000148135 | TTGACACTGTCAACAATTAGC | GE Dharmacon |
| shSTAG2-1 | TRCN0000151221 | AAACAAAGCTGTAAGAACTGC | GE Dharmacon |
| shSTAG2-2 | TRCN0000153782 | ATTTCGGTAAGACATCAGTGG | GE Dharmacon |
| shMYCN-1 | TRCN0000020696 | AGTAGAAGTCATCTTCGTCCG | GE Dharmacon |
| shMYCN-2 | TRCN0000020697 | ATGTTGTGGTTTCTGCGACGC | GE Dharmacon |
| | | | |
| pMD2.G | | | From Dr. Trono |
| psPAX2 | | | From Dr. Trono |
| pCBASce | | | From Dr. Jasin and Dr. Kohno |
| CSII-CMV-mRFP | | | From Dr. Miyoshi |
| MYCN-Halo | pFN21AB8396 | | Promega (Kazusa DNA Res. Inst.*) |
| control-Halo | G6591 | | Promega |

*Nagase et al., DNA Res. 15: 137-49 (2008)

Supplemetal Table 4

Primers

| | name | No. | sequence |
|---|---|---|---|
| for mice cDNA | Sgo1-F | 8 | GGTGAGGATCCCTTCTGTCA |
| | Sgo1-R | 9 | TGAGGTCCACGACAGTGCTA |
| | gapdh-F | 551 | GGTGGTGAAGCAGGCATCTG |
| | gapdh-R | 552 | GGAGGCCATGTAGGCCATGA |
| for human cDNA | SGO1-F | 481 | TCTGGAATGGACCCCAATAG |
| | SGO1-R | 482 | TGCTGGGCTTGCTTTATTCT |
| | E-box1-F | 475 | TGTCTTCGCAGCAGGACTAA |
| | E-box1-R | 476 | TAGCAGGGGGTGTGGTAGAC |
| | E-box2-F | 473 | GAGTTGTTGGCTGGAGAAGG |
| | E-box2-R | 474 | TCAGCATCTCGTTGCAATTT |
| | E-box3-F | 221 | TTCCTCTTTCAGGGACTCCA |
| | E-box3-R | 222 | GAGAGCTTCGAAGAGCCTTG |
| | E-box4-F | 223 | CTACCCTGGCCAAAGATGAA |
| | E-box4-R | 224 | TTGCCTAGCCCTAGCAATAGTT |
| | 20kb upstream-F | 231 | TCATCTAGGAGATGGGATTGG |
| | 20kb upstream-R | 232 | TACCCAGCATTTCCAGGTGT |
| | SMC1A-F | 516 | TGCCTTGGATAACACCAACA |
| | SMC1A-R | 517 | TCGAAGGTCAGGACTTTGCT |
| | SMC3-F | 520 | GGAGGGCAGTCAGTCTCAAG |
| | SMC3-R | 521 | AGCAAGGGCTACCAAGGATT |
| | RAD21-F | 510 | TGACTTTGATCAGCCACTGC |
| | RAD21-R | 511 | TCTCACGATCATCCATTCCA |
| | STAG2-F | 524 | TGCTATGCAGTCGGTGGTAG |
| | STAG2-R | 525 | AGGACCAGCCATGGTAAGTG |
| | MYCN-F | 596 | CGACCACAAGGCCCTCAGTA |
| | MYCN-R | 597 | CAGCCTTGGTGTTGGAGGAG |
| | BRCA1-F | 271 | CTCCCTGTTGCTGAAACCAT |
| | BRCA1-R | 272 | AGTCTTCACTGCCCTTGCAC |
| | BRCA2-F | 598 | GCCACCACCACACAGAATTC |
| | BRCA2-R | 599 | TTTGCTTCAAACTGGGCTGA |
| | p16-F | 547 | GAAGGTCCCTCAGACATCCCC |
| | p16-R | 548 | CCCTGTAGGACCTTCGGTGAC |
| | p21-F | 580 | CACCCTAGTTCTACCTCAGGCA |
| | p21-R | 581 | ACTCCCCCATCATATACCCCT |
| | GAPDH-F | 453 | ATCAATCCCTGCCTCTACTGG |
| | GAPDH-R | 454 | CCCTCCGACGCCTGCTTCAC |
